# Experiments and Calculation on New N,N-*bis*-Tetrahydroacridines

**DOI:** 10.3390/molecules29174082

**Published:** 2024-08-28

**Authors:** Madalina-Marina Hrubaru, Constantin Draghici, Francis Aurelien Ngounoue Kamga, Elena Diacu, ThankGod C. Egemonye, Anthony C. Ekennia, Eleonora-Mihaela Ungureanu

**Affiliations:** 1 “C. D. Nenitzescu” Institute of Organic and Supramolecular Chemistry, Romanian Academy, Bucharest, Sector 6, Splaiul Independentei 202B, P.O. Box 35-108, 060023 Bucharest, Romania; madalina.hrubaru@gmail.com (M.-M.H.); cst_drag@yahoo.com (C.D.); 2 Faculty of Chemical Engineering and Biotechnologies, National University of Science and Technology Politehnica Bucharest, 1-7 Polizu Street, Sector 1, 011061 Bucharest, Romania; 3 Coordination Chemistry Laboratory, Department of Inorganic Chemistry, Faculty of Science, University of Yaounde, Yaounde P.O. Box 812, Cameroon; fr.kamga@gmail.com; 4 Doctoral School Chemical Engineering and Biotechnologies, National University of Science and Technology Politehnica Bucharest, 1-7 Polizu Street, Sector 1, 011061 Bucharest, Romania; 5 Department of Pure and Applied Chemistry, Faculty of Physical Sciences, University of Calabar, Calabar 540281, Cross River State, Nigeria; egemonyethankgod200@gmail.com; 6 Department of Chemistry, Alex Ekwueme Federal University, Ndufu-Alike, P.M.B. 1010, Abakiliki 482131, Ebonyi State, Nigeria; chemisttony@gmail.com

**Keywords:** 7,7′-(ethane-1,2-diyl)*bis*(2,3-dihydro-1H-cyclopenta[b]quinoline-9-carboxylic acid), 7,7′-(ethane-1,2-diyl)*bis*(1,2,3,4-tetrahydroacridine-9-carboxylic acid), voltammetric methods, computations

## Abstract

Tetrahydroacridines arouse particular interest due to the potential possibilities of application in the medical field and protection against corrosion. *Bis*-tetrahydroacridines were newly synthesized by Pfitzinger condensation of 5,5′-(ethane-1,2-diyl) diindoline-2,3-dione with several cyclanones. NMR, MS, and FT-IR were used to prove their molecular structure. In addition, a computer-aided study was performed for the lowest energy conformers of each structure, in vacuum conditions, at ground state using DFT models to assess their electronic properties. UV–Vis and voltammetric methods (cyclic voltammetry, differential pulse voltammetry, and rotating disk electrode voltammetry) were used to investigate their optical and electrochemical properties. The results obtained for these π-conjugated heteroaromatic compounds lead to the conclusion that they have real potential in applications in different fields such as pharmaceuticals and especially as corrosion inhibitors.

## 1. Introduction

Acridines and their homologous derivatives can be found in both natural plants and marine organisms, forming an important class of heterocycles containing nitrogen in their molecule. They have been extensively studied in terms of their synthesis [1,2,3], physicochemical properties [4,5,6,7], and structural requirements [8]. However, they have also been the subject of considerable investigations regarding their possible applications [9,10,11,12,13].

Following their discovery, acridines were initially utilized as pigments and dyes [14]. Subsequently, they have been extensively investigated as potential pharmaceutical agents for the treatment of a range of illnesses, including cancer [15], Alzheimer’s disease [16,17,18], bacterial [19] and protozoan infections [20]. Their mode of action is mainly attributed to intercalation into DNA and RNA strands through the formation of hydrogen bonds and their stacking between base pairs, resulting in DNA crosslinks and strand breaks [20]. These bonds have subsequent effects on biological processes related to DNA and its associated enzymes [21,22]. Tetrahydroacridines, the reduced homologs of acridines, have received increased attention due to their ability to inhibit topoisomerase enzymes and block DNA transcription [23,24,25,26,27]. They have been widely explored for the treatment of Alzheimer’s disease [28,29,30,31,32,33,34,35,36,37,38], human cancer, [39,40,41], and tuberculosis [42]. Furthermore, new natural and synthetic acridine derivatives have been tested for antimalarial and antileishmanial [43,44,45,46,47], antifungal [48,49], antiparasitic [50], anti-inflammatory [51,52,53,54,55,56,57,58,59,60], and analgesic [61,62,63] activities, with some derivatives approved for chemotherapy [64]. Additionally, through sustained efforts, acridine derivatives with increased therapeutic potency and selectivity have been developed, such as fluorescent materials [65,66,67]. The presence of an aromatic ring, a π-system, and a nitrogen atom with an available electron pair in the chemical structure of acridines and tetrahydroacridines renders them suitable as corrosion inhibitors [68] and metal chemosensors [69,70,71].

Similarly to other π-conjugated organic molecules, acridines have been employed in several other leading fields due to their strong electron-donating capacity [72] and remarkable optoelectronic properties [73,74,75,76,77]. These include organic electronics [78,79], as well as the field of organic light-emitting diodes [80,81,82,83]. In addition, their partially hydrogenated analogs have been used as electron donor groups for OLED applications [84,85].

The present paper presents the results of the studies initiated in our group on the synthesis, electrochemical, and optical properties of tetrahydroacridine dimers 7,7′-(ethane-1,2-diyl)*bis*(2,3-dihydro-1H-cyclopenta[b]quinoline-9-carboxylic acid) and 7,7′-(ethane-1,2-diyl)*bis*(1,2,3,4-tetrahydroacridine-9-carboxylic). This expands the range of tetrahydroacridines explored so far by our research group [86,87]. The study led to the extension of their useful properties for new applications, complementing the information on the optoelectrical properties of the above tetrahydroacridines.

## 2. Results

### 2.1. Synthesis and Physical-Chemical Characterization

**Scheme 1:**
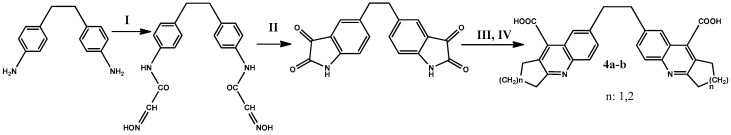
Synthesis of tetrahydroacridines analogous with 4,4′-(ethane-1,2-diyl)dianiline skeleton; I. CCl_3_-CHO, NH_2_OH, Na_2_SO_4_, T = 95–100 °C; II. H_2_SO_4_, T = 70 °C; III. KOH alcoholic solution, cyclanone (cyclohexanone or cyclopentanone for **4a** and **4b**, respectively).

For the synthesis of the new tetrahydroacridine dimers, the synthesis strategy was in accordance with the methods developed by Bielavsky [88] and Carje [89], but with some modifications imposed by the need to obtain dimers [90,91]. The overall synthesis is shown in Scheme 1.

Starting from 4,4′-(ethane-1,2-diyl)dianiline (**1**), chloral and hydroxylamine, *bis*-isonitrosoacetanilide (**2**) were prepared by Sandmayer reaction (step I) in low yield (30%). In step II of the reaction, compound (**2**) was heated at 87–95 °C in H_2_SO_4_ for twenty minutes, to obtain the corresponding *bis*-indoline-tetraone (**3**) by cyclocondensation in good yields. In step III, the corresponding *bis*-acids 7,7′-(ethane-1,2-diyl)*bis*(2,3-dihydro-1H-cyclopenta[b]quinoline-9-carboxylic acid) (**4a**) and 7,7′-(ethane-1,2-diyl)*bis*(1,2,3,4-tetrahydroacridine-9-carboxylic acid) (**4b**) were obtained by Pfitzinger condensation of *bis*-isatine (**3**) with cyclanones in a solution of KOH in ethanol. The final compounds have low solubility in organic solvents and melting points above 240 °C with decomposition [90,91]. In the following paragraphs, the specific working conditions for the compounds resulting from each step, the yields of the steps, and the spectral data for the newly synthesized compounds are presented.

#### 2.1.1. N,N′-(4,4′-(Ethane-1,2-diyl)*bis*(4,1-phenylene))*bis*(2-(hydroxyimino)acetamide) (**2**)

**Figure 1:**
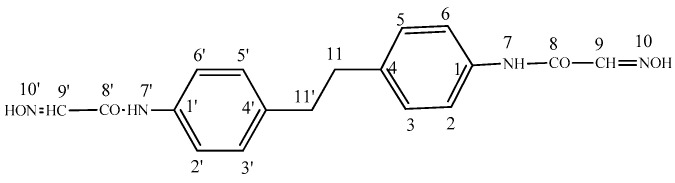
Molecular structure of N,N′-(4,4′-(ethane-1,2-diyl)*bis*(4,1-phenylene))-*bis*(2-(hydroxyimino) acetamide) (**2**).

Chloral hydrate (0.22 mol, 32.5 g) was dissolved in 1000 mL water with Na_2_SO_4_ (1.60 mol, 230 g). The solution was heated at 60 °C before the solution of hydroxylamine hydrochloride (0.22 mol in 100 mL water) was added. In a separate flask, a slurry of 4,4′-(ethane-1,2-diyl)dianiline (0.1 mol, 21.2 g), in 120 mL water with 17.3 mL concentrated HCl was prepared. The two reaction mixtures were combined, and the reaction temperature was increased to 90–95 °C for 20 min. After that, the mixture was cooled in an ice bath for 15 min. The precipitate was collected by filtration, recrystallized in C_2_H_5_OH, filtrated off, and poured into a cold solution of diluted ammonia. The yellowish precipitate was separated by filtration, washed with water (30 mL) and C_2_H_5_OH, and dried. The yield of the title compound (Figure 1): 85% (30.12 g); M.p.: over 240 °C. Anal. calcd. for C_18_H_18_N_4_O_4_ (354.4): C 61.01, H 5.12, N 15.81. Found: C 60.95, H 5.01, N 15.90; FT-IR(solid, ATR, cm^−1^): 3315 m, 2940, 2860 m, 1639 s, 1621 s, 1600 vs, 1545 vs, 1514 m, 1464 m, 1414 m, 868 w, 829 m; ^1^H-NMR (DMSO-d6, δ ppm, *J* Hz):12.13(s, 2H, H10, H10′); 10.08(s, 2H, NH, H7, H7′); 7.65(s, 2H, H9, H9′); 7.57(d, *J* = 8.4, 4H, H2, H2′, H6, H6′); 7.15 (d, *J* = 8.4, 4H, H3, H5, H3′, H5′); 2.82(s, 4H, H11, H11′); ^13^C-NMR(DMSO-d6, δ ppm): 160.04(C8, C8′); 144.11(C9, C9′); 137.02(C1, C1′); 136.27(C4, C4′); 128.34(C3, C3′,C5, C5′); 119.8(C2, C2′, C6, C6′); 36.50(C11, C11′); Negative ESI-MS (*m*/*z*): 353 [M − H]^−^, 308 [M − H − CH_2_NOH]^−^, 263 [M − H − 2CH_2_NOH]^−^, 176 [M/2 − H]^−^, 132 (M/2 − H − CH_2_NO]^−^.

#### 2.1.2. 5,5′-(Ethane-1,2-diyl)diindoline-2,3-dione (**3**)

**Figure 2:**
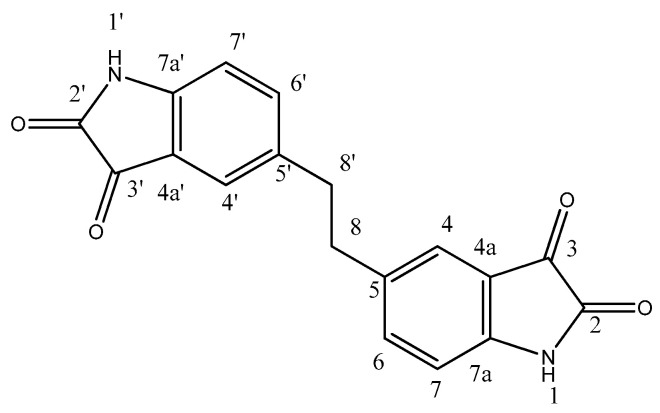
Molecular structure of 5,5′-(ethane-1,2-diyl)diindoline-2,3-dione (**3**).

N,N′-(biphenyl-4,4′-diyl)*bis*(2-(hydroxyimino)acetamide derivative (**2**) (0.03 mol, 10.62 g) was added in small portions, for 8–10 min, to a solution of sulfuric acid (0.72 mol, 44.14 mL, 88.3%, d = 1.81) at 70–72 °C, under vigorous stirring. The temperature was maintained at 87–95 °C for 30 min to complete the reaction. The mixture was cooled at room temperature and poured into a mixture of ice and water. The resulting crude product was stirred for 30 min, filtered off, and washed several times with water to neutral pH. A deep-purple powder was obtained. The *bis*-isatine (**3**) was purified by dissolving it in an aqueous alkali of 5%, neutralizing it with dilute acetic acid, filtering off the separated dark material, and acidifying the solution with hydrochloric acid. Then, the solution was cooled rapidly, and the *bis*-isatine (Figure 2) was filtered off. Yield 82% (7.87 g); M.p.: over 240 °C; Anal. calcd. C_18_H_12_N_2_O_4_ (320.30): C 67.50, H 3.78, N 8.75. Found: C 67.61, H 3.83, N 8.87; IR(solid, ATR, cm^−1^): 3255 s, 2860 m, 1723 s, 1704 s, 1482 m; ^1^H-NMR (DMSO-d6, δ ppm, *J* Hz): 10.95(s, 2H, NH, H1, H1′); 7.43(dd, *J* = 1.9, *J* = 8.0, 2H, H6, H6′); 7.38(d, *J* = 1.9, 2H, H4, H4′); 6.81(d, *J* = 8.0, 2H, H7, H7′); 2.79(s, 4H, H8, H8′); ^13^C-NMR (DMSO-d6, δ ppm): 184.71(C3, C3′); 159.64(C2, C2′); 149.04(C7a, C7′a); 138.66(C4, C4′); 136.07(C4a, C4′a); 124.69(C6, C6′); 117.90(C5, C5′); 112.19(C7, C7′); 36.05(C8, C8′). Negative ESI-MS: (*m*/*z*): 319 [M − H]^−^; 291 [M − H − CO]^−^; 263 [M − H − 2CO]^−^;248 [M − H − 2CO − 1/2N_2_H_2_]^−^; 220 [M − H − 3CO − 1/2N_2_H_2_]^−^; 159 [M/2 − 2H]^2−^.

The general procedure for the synthesis of corresponding dicarboxylic acids (**4a-b**) is the following: 0.1 mol of 5,5′-(ethane-1,2-diyl)diindoline-2,3-dione (**3**) is dissolved in 0.3 mol of KOH (30% solution) in 100 mL of C_2_H_5_OH. Over this mixture, 0.3 mol of cyclanones was added with stirring. The reaction mixture was stirred at reflux for 12 h. Then, the solution was concentrated to half volume by vacuum distillation, and the resulting precipitate was filtered off by vacuum and washed several times with acetone and ethyl ether. After cooling, 0.34–0.4 mole of CH_3_COOH (50%) was added to the remaining solution. The solid obtained was purified by several successive recrystallizations from alcohol/CH_3_CN. The melting points of the compounds are above 240 °C with decomposition.

#### 2.1.3. 7,7′-(Ethane-1,2-diyl)*bis*(2,3-dihydro-1H-cyclopenta[b]quinoline-9-carboxylic Acid) (**4a**)

**Figure 3:**
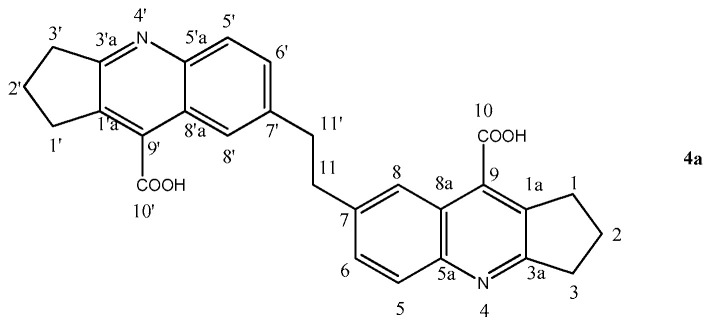
Molecular structure of 7,7′-(ethane-1,2-diyl)*bis*(2,3-dihydro-1H-cyclopenta[b]quinoline-9-carboxylic acid) (**4a**).

According to the general procedure, 0.1 mol of 5,5′-(ethane-1,2-diyl)diindoline-2,3-dione) (**3**), KOH (0.3 mol, 16.83 g) (30% solution) and cyclopentanone (0.3 mol, 25.24 g) provided 18.55 g of the title compound (Figure 3).

Yield 41% (18.55 g); M.p.: over 240 °C Anal. calcd. C_28_H_24_N_2_O_4_ (452.51): C 74.32, H 5.35, N 6.19. Found: C 75.11, H 5.48, N 6.25; IR(solid, ATR, cm^−1^): 3359 m, 2949 m, 2881 m, 1699 vs, 1594 vs, 1510 w, 1427 w, 1224 s, 886 w, 823 m, 664 w; ^1^H-NMR (DMSO-d6+ TFA, δ ppm, *J* Hz): 8.07(d, 2H, *J* = 8.8, H5, H5′); 7.91(dd, *J* = 8.8, *J* = 1.6, 2H, H6, H6′); 7.86(d, *J* = 1.6, 2H, H8, H8′); 3.40(t, *J* = 7.2, 4H, H3, H3′); 3.18(t, *J* = 7.4, 4H, H1, H1′); 2.50(s, 4H, H11, H11′); 2.16(cv, *J* = 8.0, 4H, H2, H2′); ^13^C-NMR (DMSO-d6 + TFA, δ ppm): 165.80(C10, C10′); 165.70(C9, C9′); 142.42(C3a, C3′a); 140.60(C5a, C5′a); 137.34(C7, C7′); 136.36(C1a, C1′a); 134.24(C8, C8′); 124.98(C6, C6′); 123.67(C8a, C8′a); 120. 68(C5, C5‘); 36.49(C3, C3′); 31.82(C11, C11′); 30.01(C1, C1′); 22.75(C2, C2′). Positive ESI-MS *m*/*z*: 453 [M + H]^+^; 409 [M + H − CO_2_]^+^; 365 [M + H − 2CO_2_]^+^; 183 [M + 2H − 2CO_2_]^2+^. Negative ESI-MS *m*/*z*: 451 [M − H]^−^; 407 [M − H − CO_2_]^−^; 363 [M/2 − H − 2CO_2_]^−^; 182 [M/2 − 2H − 2CO_2_]^−^. UV (λ_max_, nm): 270, 311, 325.

#### 2.1.4. 7,7′-(Ethane-1,2-diyl)*bis*(1,2,3,4-tetrahydroacridine-9-carboxylic Acid) (**4b**) 

**Figure 4:**
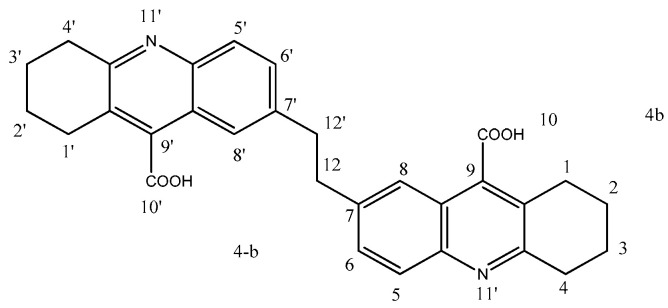
Structure of 7,7′-(ethane-1,2-diyl)*bis*(1,2,3,4-tetrahydroacridine-9-carboxylic acid) (**4b**).

According to the general procedure, 0.1 mol of 5,5′-(ethane-1,2-diyl)diindoline-2,3-dione) (**3**), KOH (0.3 mol, 16.83 g) (30% solution), and cyclohexanone (0.3 mol, 29.44 g), provided 19.7 g of the title compound (Figure 4).

Yield 41% (19.7 g); M.p.: over 240 °C Anal. calcd. C_30_H_28_N_2_O_4_ (480.56), C, 74.98; H, 5.87; N, 5.83. Found: C 75.2, H 5.13, N 5.24; IR(solid, ATR, cm^−1^): 3485 s, 3367 s, 2941 s, 2865 s, 1590 s, 1397 s, 1369 m; ^1^H-NMR (DMSO-d6 + TFA, δ ppm, *J* Hz): 8.14(d, 2H, *J* = 8.8, H5, H5′), 8.03(dd, 2H, *J* = 1.7, *J* = 8.8, H6, H6′), 7.57(d, 2H, *J* = 1.7, H8, H8′), 3.29(t, 4H, *J* = 6.3, H4, H4′), 2.91(t, 6.2, 4H, H1, H1′), 2.50(s, 4H, H12, H12′)1.95–1.84(m, 8H, H2, H3, H2′, H3′); ^13^C-NMR (DMSO-d6 + TFA, δ ppm): 166.47(C10, C10′), 157.72(C9, C9′), 146.55(C4a, C4′a), 142.76(C5a, C5′a), 135. 64(C1a, C1′a), 135.35(C8, C8′), 128.02(C7, C7′), 124.09 (C6, C6′), 36.40(C12, C12′); 28.81(C1, C1′); 25.40(C4, C4′); 20.68(C2, C2′); 20.16(C3, C3′); Positive ESI-MS *m*/*z*: 481 [M + H]^+^; 393 [M + H − 2CO_2_]^+^; 197 [M/2 + 2H − 2CO_2_]^2+^; Negative ESI-MS *m*/*z*: 479 [M − H]^−^; 435 [M − H − CO_2_]^−^; 391 [M − H − 2CO_2_]^−^; 195 [M/2 − 2H − 2CO_2_]^2−^, UV (λ_max_, nm): 268, 311, 326.

### 2.2. Computations

**Figure 5:**
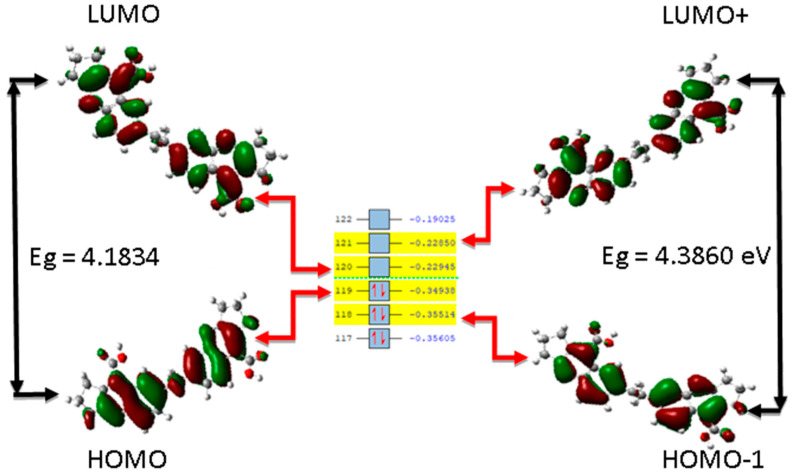
HOMO, HOMO-1, LUMO, LUMO + 1 plots for compound **4a**.

**Figure 6:**
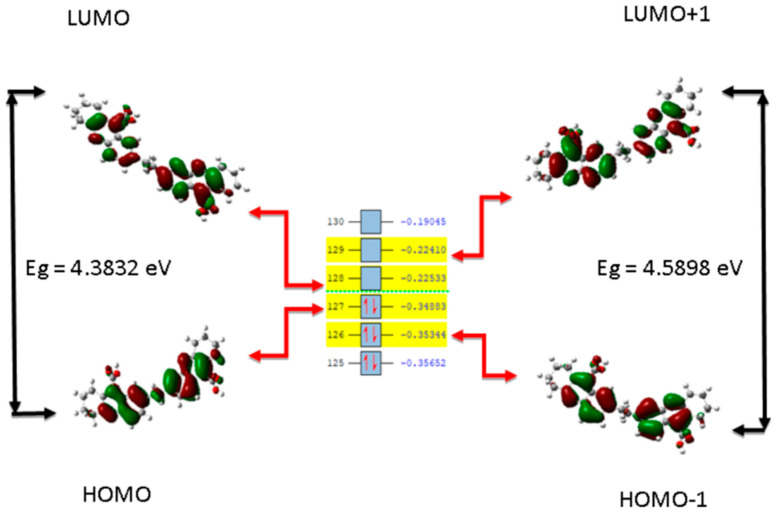
HOMO, HOMO-1, LUMO, LUMO + 1 plots for compound **4b**.

Predictions based on the electronic structure theory for **4a** and **4b** tetrahydroacridines were completed using the density functional theory (DFT) method. Geometry optimization of the structure was performed on model structures and outputs used for further geometry optimization. The highest occupied molecular orbital (HOMO), and the lowest unoccupied molecular orbital (LUMO), which are the orbitals that constitute the frontier molecular orbitals (FMO), were energetically evaluated to comprehend the nature of reactivity and stability of the studied compounds according to previous studies [92]. All FMO (HOMO and LUMO given in Table 1) isosurface maps were plotted. Starting from their calculated energy values, quantum reactivity parameters were calculated: chemical potential (µ), ionization potential (I), electron affinity (A), electronegativity (χ), hardness (η), softness (σ), as listed in Table 1. Also, from the FMOs analysis, the energy gap (Eg) value was obtained as the difference between the HOMO and LUMO energy levels. The results are shown in Figure 5 and Figure 6 for **4a** and **4b**, respectively, and agree with other studies [93]. A higher Eg value signifies more stability and less reactivity, while a low Eg value indicates less stability and high reactivity [94].

The natural bond orbital (NBO) data of **4a** and **4b** are given in Table 2 and Table 3. NBO analysis was conducted on the investigated compounds to bring to light the type of interaction occurring between the donor orbital and the acceptor orbital [95]. NBO of the studied compounds was completed to gain insights into the intramolecular and intermolecular hyperconjugation, electron delocalization, and intermolecular charge transfer (ICT) [96] persisting within the molecules. The nature of the interactions existing between the donor orbital and acceptor orbital is expressed in terms of second-order perturbation energy (E^(2)^) or stabilization energy (E^(2)^) [97]. The higher the stabilization energy, the stronger the interaction between the donor orbital and the acceptor orbital. The second-order perturbation energy of the studied compounds was estimated [98].

Fukui function, a quantum chemical approach proposed by Kenchi Fukui to describe the change in electron density of a particular atom within a molecule with respect to the change in the number of electrons present [99] was also examined. Condensed Fukui function was used as a form of local reactivity descriptor to provide better information on the nature of the reaction taking place at a preferential site of an atom within a molecule such as nucleophilic attack or electrophilic attack [100]. Thus, this quantum chemical approach of great importance in organic synthesis for studying electrophilic and nucleophilic reactions [101] was used.

### 2.3. Electrochemistry

The electrochemical behavior of the two *bis*-acids **4a** and **4b** was studied by several electrochemical techniques such as cyclic voltammetry (CV), differential pulse voltammetry (DPV), and rotating disk electrode voltammetry (RDE). Due to the low solubility of **4a** and **4b** in acetonitrile, the study was performed in dimethylformamide containing 0.1 M tetrabutylammonium perchlorate (TBAP) as a supporting electrolyte. The study was conducted on clean glassy carbon (GC) electrodes in the potential range of −3 V to +3 V vs. Ag/Ag^+^ reference electrodes. Each scan started from the stationary potential. After the experiments, the potential axis was referred to the redox couple ferrocene/ferrocenium (Fc/Fc^+^), and the resulting curves were shown consequently. The peaks were denoted in the order of their appearance in DPV and CV direct scans. However, their quantification and specifying the peak potentials for dimers **4a** and **4b** proved to be much more complicated compared to the electrochemical characterization of the monomers with a similar structure previously studied [87]. In all DPV and CV scans, the peaks were established with difficulty through the correlations between the characteristics of the CV and DPV curves.

#### 2.3.1. Electrochemical Study of **4a** *bis*-Acid

**Figure 7:**
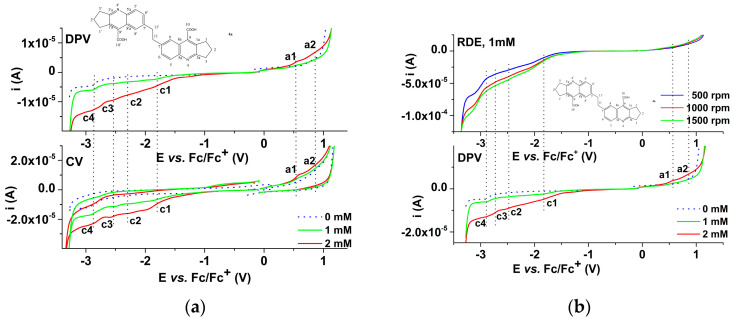
DPV vs. CV (0.1 V/s) (**a**) and RDE vs. DPV (**b**) curves at different concentrations of **4a**.

**Figure 8:**
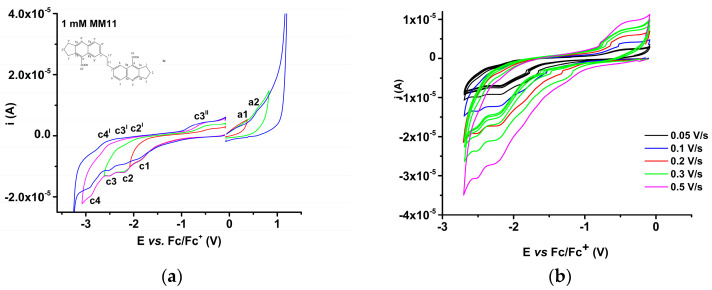
CV curves in anodic and cathodic scans on variable domains at 0.1 V/s (**a**) and at variable scan rates in 5 successive CV (0.1 V/s) cathodic scans (**b**) for [**4a**] = 1 mM.

**Figure 9:**
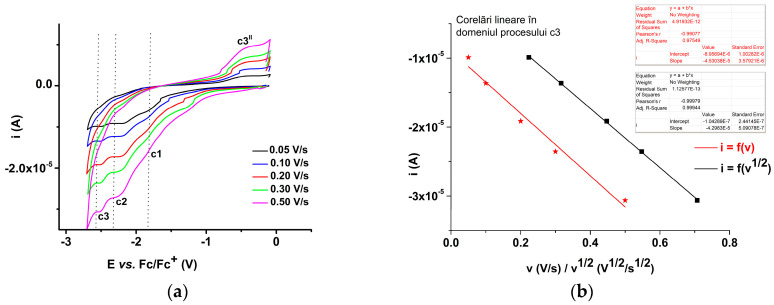
CV curves (0.1 V/s) in the first cathodic scans at variable scan rates (**a**) and the dependence of current intensity at −2.6 V on CV curves (in the 1st cycle) on scan rate (red line and points) or on the square root of the scan rate (black line and points) (**b**) for [**4a**] = 1 mM.

**Figure 10:**
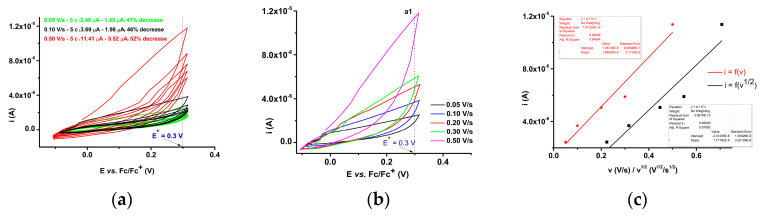
CV curves in the first 5 cycles of anodic scans at variable scan rates (**a**), the first cycle at variable scan rate (**b**), and the dependence of current intensity at 0.3 V on CV curves (in the 1st cycle) on the scan rate (red line and points), or on the square root of the scan rate (black line and points) (**c**) for [**4a**] = 1 mM.

**Figure 11:**
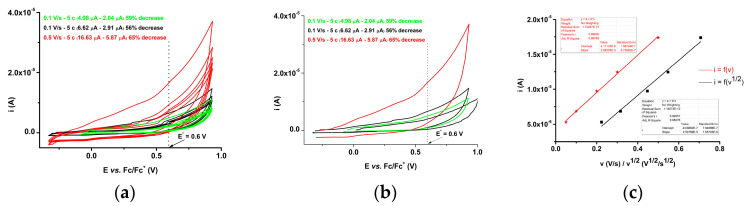
CV curves in the first anodic scans at variable scan rates (**a**), CV curves in the first anodic scan at variable scan rates (**b**), and the dependence of current intensity at 0.6 V from the 1st cycle of the CV curves on the scan rate (red line and points) or on the square root of the scan rate (black line and points) (**c**) for [**4a**] = 1 mM.

**Figure 12:**
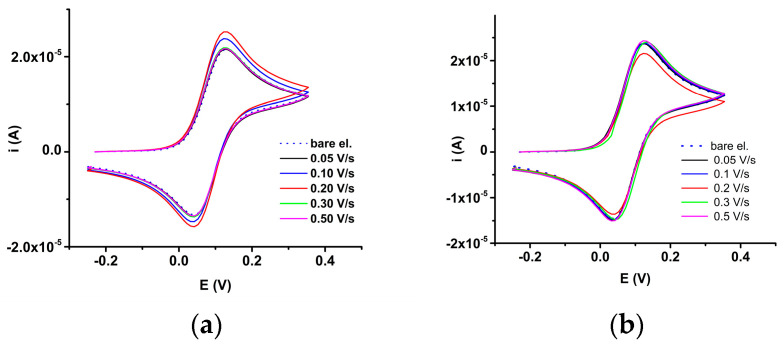
CV curves (0.1 V/s) in ferrocene solution in 0.1 M TBAP/CH_3_CN on bare GC electrode (...) and on the electrode after 5 cycles in the range of a1 process (**a**) or a2 process (**b**) in solution of [**4a**] = 1 mM.

Selected anodic and cathodic DPV, CV, and RDE curves connected to the electrochemical behavior of **4a** are shown in Figure 7, Figure 8, Figure 9, Figure 10, Figure 11 and Figure 12. The peak potentials obtained by DPV method for **4a** are shown in Table 4. 

#### 2.3.2. Electrochemical Study of **4b** *bis*-Acid

**Figure 13:**
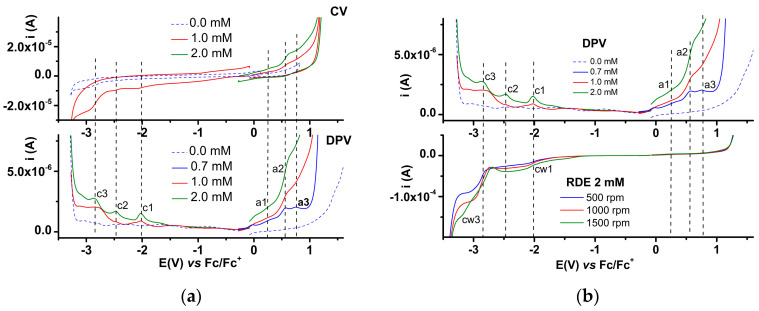
CV (0.1 V/s) vs. DPV (**a**) and DPV vs. RDE (**b**) curves at different concentrations of **4b** and variable electrode rotation rates.

**Figure 14:**
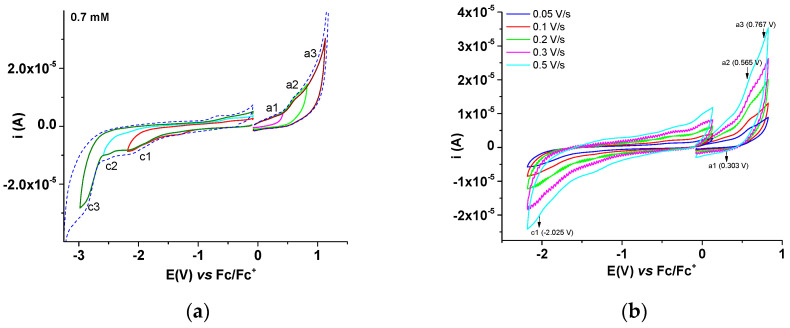
CV curves (0.1 V/s) in anodic and cathodic scans on variable domains (**a**) and at variable scan rates in 5 successive cathodic and anodic scans (**b**) for [**4b**] = 1 mM.

**Figure 15:**
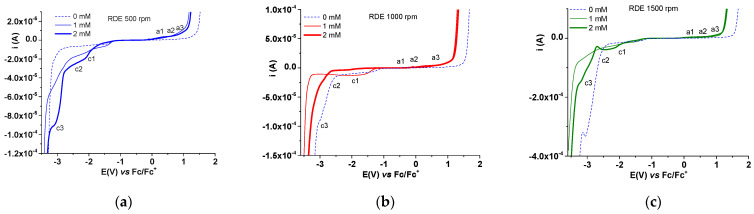
Anodic and cathodic RDE curves at different concentrations of **4b** at various rotation rates: 500 rpm (**a**), 1000 rpm (**b**), and 1500 rpm (**c**).

The analysis of the behavior of compound **4b** was conducted in a similar way as in the case of **4a**. The main results are shown in Figure 13, Figure 14 and Figure 15, and in Table 5 and Table 6, which are discussed further.

## 3. Discussion

**Synthesis** of *bis*-acids7,7′-(ethane-1,2-diyl)*bis*(2,3-dihydro-1H-cyclopenta[b]quino-line-9-carboxylic acid) (**4a**) and 7,7′-(ethane-1,2-diyl)*bis*(1,2,3,4-tetrahydroacridine-9-carboxylic acid) (**4b**) was performed in three steps consisting of a Sandmayer reaction (step I) followed by a cyclization in acidic medium with the formation of *bis*-indoline tetraone (step II), and a Pfitzinger cyclization with a cyclanone (step III). Stage I proceeded with lower yields than in the case of the corresponding monomers (30%), due to the intermediate formation of hydrochloride at one of the amino groups. However, the synthesis of the corresponding intermediate succeeded, and it was transformed during stage II with good yields (82%) and stage III with moderate yields (41%).

The characterization of compounds involved in the synthesis of the two new tetrahydroacridine dimers **4a** and **4b** has proven the formation of title compounds. The ^1^H-NMR and ^13^C-NMR spectra were indicative of the compounds’ formation. The dimers have low solubility in common organic solvents. Their melting points are above 240 °C with decomposition. The final compounds were also characterized by their UV–Vis, FT-IR, and MS spectra. Their structures were calculated using DFT methods and their electrochemical behavior was examined.

The **^1^H-NMR spectra** of tetrahydroacridine *bis*-acids, **4a**–**b**, show the two distinct sub-spectra of the aromatic ring and the saturated ring. Following cyclocondensation of *bis*-isatines (3) with cyclanones to *bis*-tacrinacids, aromatic protons retain their multiplicity, but their chemical shifts are significantly modified, resonating at lower values of the field due to the deshielding produced by the current ring, the electronic effect of the quinoline nitrogen atom and the carboxylic group. The most deshielded are the vicinal protons with the quinoline nitrogen (H-5, H-5′), which register a deshielding of approximately δ = 0.8–1 ppm and appear as a doublet at δ = 7.81–8.14 ppm. The protons with the same multiplicity (H-8, H-8′) appear as a doublet at δ = 7.52–7.86 ppm. The protons H-6 and H-6′ are more shielded and resonate as a doublet of a doublet at δ = 7.62–8.02 ppm. The deshielding effect of the carboxylic group on the methylene groups of the saturated cycles is also observed, with these groups resonating in the range of δ = 3.01–3.32 ppm. The most deshielded of these groups are those placed in the adjacent position with the acridinic nitrogen, followed by those placed in the neighboring position of the carboxylic group. Following cyclocondensation, the protons of the ethylene bridge, -CH2-CH2-, are also deshielded and undergo a chemical displacement of approximately 0.3 ppm, reaching δ = 3.1–3.2 ppm in *bis*-acids.

In the **^13^C-NMR spectra** of the tetrahydroacridine *bis*-acids, the most deshielded signals are assigned to the carboxylic groups that resonate in the range δ = 165.80–166.47 ppm and to the vicinal carbons found in the γ position by the acridinic nitrogen C9, C9′, which resonate in the field δ = 157.91–164 ppm. In contrast to *bis*-isatines, the tertiary carbon atoms exhibit minimal displacement. The saturated cycles of *bis*-tacrinacids manifest as methylene group signals at δ = 25–30 ppm, with the most distorted ones being those nearest to the carboxyl groups. For the ethylene group, the chemical shifts are maintained within the range δ = 37–38 ppm.

The **UV spectra** of *bis*-acids present 3 bands of maximum absorption corresponding to a π → π* transition at 268/270 nm and corresponding to a n → π* at 311 nm and 325/326 nm in DMF solutions for **4a**/**4b** compounds, respectively (Figures S1 and S2). Their absorbances depend on concentration linearly (Table S1). From the comparison of their UV–Vis spectra, **4a** is almost two times more active in absorption than **4b** (Figures S1 and S2) and the slope of absorbance confirms these facts (Table S1).

**Scheme 2:**
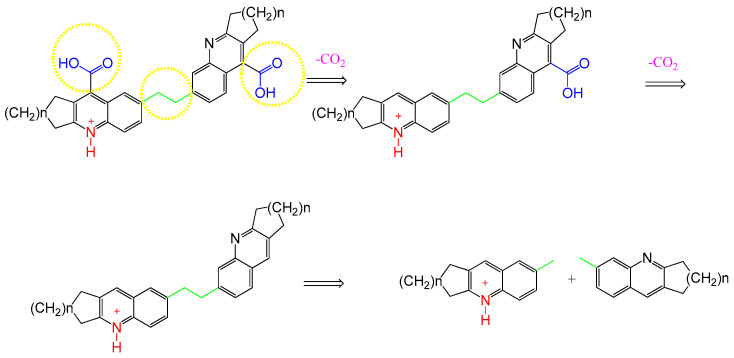
Fragmentation pattern for ethylene bridged *bis*-acids.

The **mass spectra** (MS) played an important role in elucidating the structure of the new compounds. The molecular peaks of the ions formed by fragmentation are indicated for each compound in Scheme 2. Ionization of the ethylene *bis*-acids presented in this study, as well as other *bis*-amides/*bis*-acids previously presented [91], occurs with the formation of double ions of the [M + 2H]^2+^ type having *m*/*z* values corresponding to half of the value for the dimer structure, and an abundance much lower than that of the protonated molecular ions [M + H]^+^. Further, the double ions [M + 2H]^2+^ lose functional groups or are fragmented at the ethylene bridge with the appearance of ions of half molecule type, [M/2]^+^. The acids are also ionized in negative mode using electrospray, when a solvent slightly basic is used, such as methanol/ water with 0.5% ammonium carbonate. In positive mode, ionization of ethylene *bis*-polymethylenquinolin *bis*-acids occurs easily by the successive elimination of one carbon dioxide molecule.

The **IR (solid ATR) spectra** given for each newly synthesized compound highlight the characteristic functional groups and skeleton frequencies. In the precursors (*bis*-isonitrosoacetanilide and *bis*-isatine) the amidic groups show for the NH group, a stretching vibration ν(NH) in the field 3100–3350 cm^−1^ and a deformation vibration δ(NH) at 1530–1580 cm^−1^. In the carbonyl and azomethyin groups, the ν(C=O) and ν(C=N) vibrations within the range 1600–1680 cm^−1^ were noticed. In the diacids, the carboxyl groups are recognized both by the frequency of ν(CO) at 1695–1700 cm^−1^ as well as the frequency in the 2200–2800 cm^−1^ domains, which appear in the case of the acid dimer associations. The δ(C=N) group from the quinoline nucleus appears in the range 1600–1610 cm^−1^. The aliphatic methylene groups show symmetric and asymmetric ¥(CH) vibrations in the range 2840–2950 cm^−1^ and valence angle deformations ¥ (CH2) in the range 1300–1425 cm^−1^, while ν(=CH) frequencies appear over 300–3150 cm^−1^.

**Computation analyses** play a great role in discerning the reactivity and stability of compounds. The energy of FMO (HOMO and LUMO) given in Table 1 allowed the calculation of the energy gap (Eg) value from the difference between the HOMO and LUMO energy levels. The results indicate that compound **4a** has the lowest Eg value of 4.1834 eV compared to compound **4b** with a higher Eg value of 4.3832 eV. This indicates that compound **4a** is less stable and more reactive than compound **4b**.

NBO analysis conducted on the investigated compounds obtains insight into the nature of interactions existing between the donor orbital and acceptor orbital, which is expressed in terms of second-order perturbation energy (E^(2)^) or stabilization energy (E^(2)^) [99]. The higher the stabilization energy the stronger the interaction between the donor orbital and the acceptor orbital. The second-order perturbation energy of the studied compounds was estimated using Equation (1) [100]:

(1) $$E^{\left(2\right)}={∆E}_{i,j}={-q}_{i}\frac{F^{2}(i,j)}{\varepsilon_{i}-\varepsilon_{j}}$$

In (1) *q_i_* is the donor orbital occupancy, *ε_i_* and ε_j_ indicates the diagonal elements and F_(i,j)_ represents the Fock matrix element. Evaluated second-order perturbation energy of compounds **4a** and **4b** displayed in Table 2 and Table 3, respectively, shows that the most persisting form of donor–acceptor orbital interaction resulting from intermolecular hyperconjugation existing within the studied compounds is that of bonding interaction of which that of compound **4a** is primarily σ → σ* and π → σ* while that of compound **4b** is σ → σ* bonding interaction. From the displayed second-order perturbation energy E^(2)^ of the studied compounds, it is obvious that compound **4a** exhibited higher E^(2)^ values from the donor–acceptor interaction compared to compound **4b**. As seen from the tables, the highest donor–orbital interaction of compound **4a** was from donor (σC_42_–H_50_) to acceptor (σ*C_37_–H_43_) intermolecular hyperconjugation with stabilization energy of 75,640.17 kcal/mol while that of compound **4b** is from donor (σC_32_–O_33_) to acceptor (σ*C_42_–C_43_) with stabilization energy of 8691.80 kcal/mol. In this sense, we can infer that compound **4a** is more reactive than compound **4b** since the E^(2)^ value resulting from intermolecular hyperconjugation prevailing within the studied compound aids in defining the nature of the reactivity of the compounds. This result is also validated by the frontier molecular orbital (FMO) analysis of the studied compounds.

Fukui function used to describe the change in electron density of a particular atom within a molecule with respect to the change in the number of electrons present provided better information on the nature of the reaction taking place at a preferential site of an atom within these molecules. The condensed Fukui function of the studied compounds at N + 1, N − 1, N electron system with its condensed dual descriptor was computed using Equations (2), (3), (4) and (5), respectively.

(2) $$f^{+}=q_{N}-q_{N+1}\;\left(\mathrm{Nucleophilic}\;\mathrm{attack}\right)$$

(3) $$f^{-}=q_{N-1}-q_{N}\;\left(\mathrm{Electrophilic}\;\mathrm{attack}\right)$$

(4) $$f^{0}=q_{N-1}-q_{N+1}\;\left(\mathrm{Free}\;\mathrm{radical}\;\mathrm{attack}\right)$$

(5) $$∆f=f^{+}-f^{-}$$

In (2)–(5) f^+^, f^−^, f^0^ are condensed Fukui functions for nucleophilic attack, electrophilic attack, free radical attack, respectively, and q_N+1_, q_N−1_, q_N_ represents the atomic charges of the atomic sites at N + 1, N − 1, N electron system, respectively. ∆f is the condensed dual descriptor (CDD). From Equation (5), if ∆f > 0 the atomic site is prone to nucleophilic attack, and if ∆f < 0 the atomic site is preferential for electrophilic attack. For compound **4a**, the order of electrophilic attack at the preferential atomic sites was found to be C4 > C9 > C12 > C1> C3 > C31 > C6 > C13 > C7 = C8 while the point of nucleophilic attack was observed on the following atoms with trend as follows: C24 > C22 > N15 > N14 > O25 > O23 > C20 > C18 > C19 > C17. Atomic sites C7 and C8 with equivalent condensed dual descriptors for electrophilicity indicate that electrophilic attacks can take place at the same rate at their atomic site. Also, for compound **4b** the rate of electrophilic attack at the preferential site is observed to be of the trend C4 > C9 > C1 > C12 > C3 > C33 > C6 > C7 > C8 > C13, while the atomic sites prone to nucleophilic attack are of the trend C24 > C22 > N15 > N14 > O25 > C19 > C17 > O23 > C18 > C20. In general, we noticed that atomic sites C4 and C24 are the most preferential sites susceptible to electrophilic and nucleophilic reactions, respectively, for both compounds **4a** and **4b** in the presence of a chemical reagent. In addition, compound **4a** exhibited higher values of ∆f or CDD values for electrophilic and nucleophilic reactions at the preferential atomic sites compared to compound **4b**. This result implies that compound **4a** is more reactive than compound **4b,** which is in excellent agreement with the frontier molecular orbital and natural bond orbital (NBO) analysis result.

Analysis of the electrochemical behavior of the dimers **4a** and **4b** was based on DPV, CV, and RDE curves recorded in different conditions.

In the case of **4a**, from Figure 7 relating to different concentrations of **4a** the anodic and cathodic currents (continuous lines) are higher than those for the supporting electrolyte (dashed lines) and increase with the concentration of **4a**. This shows that **4a** undergoes oxidation and electrochemical reduction processes. There are two main anodic processes denoted by a1 and a2 (Figure 7a). In the cathodic DPV scans, four peaks were noticed (denoted c1–c4 in Figure 7a). From Figure 7b, processes a1 and a2 in DPV do not correspond to a wave in RDE at any rotation rate of the electrode. On the other hand, in the cathodic domain, it is clearly observed that the RDE curve has limiting currents that increase with the rotation rate of the electrode. They have been studied in detail and will be discussed further. The parallel presentation of the DPV curves and the RDE curves obtained at different concentrations of **4a** at a rotation rate of 1000 rpm and the RDE curves obtained for the concentration of 1 mM and 2 mM at different rotation rates of the electrode illustrate the difference between the information provided by these methods (anodic processes appear only in DPV, while cathodic ones are revealed by both methods).

In Figure 8a, the CV curves are given on various potential domains, anodic and cathodic, which allow the evaluation of the reversibility of the highlighted electrochemical processes, which take place at different potentials. It is observed that anodic processes are irreversible, having no correspondence in the return sweep. These oxidation processes can lead to the formation of films on the surface of the electrode and have been further analyzed because they occur relatively easily (at moderate anodic potentials close to 0.6 and 0.9 V, potentials at which peaks were recorded on the DPV curves). The presence in the CV curve of the processes c2′, c3′, and c4′ highlighted at the return sweep after the cathodic scan shows that they are quasi-reversible (Figure 8a). This behavior was verified by recording CV curves at different sweep rates (between 0.05 and 0.5 V/s) in several successive cycles for each scan rate, as in Figure 8b.

For the first cathodic scans (Figure 9a), the values of the peak currents in the c3 process domain were represented as a function of the scan rate (I vs. v) or the square root of the scan rate (I vs. v^1/2^) (Figure 9b). The correlation of the current intensity with v^1/2^ is better, justifying a possible diffusive process in reduction.

Analyzing the anodic processes (Figure 10), it is observed that in the a1 process domain, the CV curves decrease in successive cycles, regardless of the scan rate (Figure 10a). Figure 10b shows the corresponding CV curves obtained in the first cycle at various scan rates. For a potential of 0.3V corresponding to the beginning of the a1 process, it is observed (Figure 10c) that the current value correlates better with the scan rate (R = 0.98) than with the square root of the scan rate (R = 0.96), supporting a process different from the purely diffusive one. A similar analysis (Figure 11) was made for the study of the dependence of the current on the scan rate at the potential of 0.6 V, at the beginning of the process denoted by a2. In this case, the linear correlations are slightly better for the dependence of the current on the scan rate (R = 0.999) than that on the square root of the scan rate (R = 0.993). The processes a1 and a2 could be related to film formation by electrochemical oxidation of **4a**. To check this hypothesis, the electrode was transferred into a ferrocene solution (Fc/Fc^+^) in 0.1 M TPAP in ACN and the CV curves were compared with those on the bare electrode. Figure 12 shows the curves obtained after the transfer in the Fc/Fc^+^ solution of the electrodes modified by cycling at different scan rates. The cycling was completed in the **4a** solution of 1mM concentration, in the process domain a1 (a) or a2 (b). The dashed line represents the curve obtained for Fc/Fc^+^ on the bare electrode. From the examination of the curves, it is noted that the potential of the Fc/Fc+ system does not change. The increase in the peak currents for process a1 could stand for the formation of a conductive film on the surface. The current variation is insignificant in the case of process a2. This may stand for different modified electrodes obtained by cycling in a2 domain than in a1.

The electrochemical behavior of **4b** is illustrated in Figure 13a, where the CV and DPV curves for the **4b** solutions are shown in parallel at different concentrations, evidencing by both methods that **4b** undergoes electrochemical oxidation and reduction processes. In Figure 13b, DPV curves are compared with RDE curves (obtained at different rotation rates for the highest concentration studied) to make the connection between the processes evidenced by these methods. The DPV peak potentials are given in Table 5. There are three main anodic processes denoted with a1–a3 and three cathodic peaks (denoted c1–c3) in Figure 13a. From Figure 13b, it is seen that the processes a1-a3 placed in evidence by CV and DPV do not correspond to any RDE wave at all RDE rotation rates. On the other hand, in the cathodic range, two RDE waves (denoted cw1 and cw3) are clearly noticed, having limiting currents that increase with the rotation rate of the electrode. A special behavior is seen for the c2 peak where the current decreases towards the value of the background current at which all the curves intersect. This behavior could only be explained by the formation of a non-conductive film at this potential.

In Figure 14a, the CV curves for **4b** are given over different anodic and cathodic potential domains, to check the reversibility of the electrochemical processes highlighted for **4b**. All anodic and cathodic processes are irreversible for compound **4b**, having no corresponding peaks in the return sweeps. This irreversibility may be due to oxidation or reduction processes with the formation of films on the electrode surface. This behavior was tested by recording CV curves at different scan rates (between 0.05 and 0.5 V/s) (Figure 14b). The variation of the anodic and cathodic currents with the scan rate and the square root of the scan rate, respectively, was examined for **4b**. The slopes of the correlation lines and the linear correlation coefficients are displayed in Table 6. For the correlations examined for processes a1-a3 and c1, it is seen that the dependence of the current on the square root of the scan rate has a better correlation coefficient than the dependence on the scan rate, which may imply a diffusive behavior at the potentials examined.

Figure 15 shows the RDE curves for different electrode rotation rates at three concentrations of **4b** (0.5 mM, 1 mM, 2 mM). The obtained curves support the hypothesis of film formation at the potential of the process c2. At this highest concentration value (2 mM) and at the highest rotation rate (1500 rpm) the most obvious current decrease occurs for the c2 process. This can be attributed to a reduction process with film formation.

## 4. Materials and Methods

### 4.1. Reagents

For synthesis, all chemicals were purchased from commercial suppliers and used without any further purification. For electrochemistry and UV–Vis spectral studies, high-purity reagents were used, such as dimethylformamide (DMF), acetonitrile (CH_3_CN), and tetra-n-butylammonium perchlorate (TBAP) from Sigma–Aldrich (Darmstadt, Germany), which were received without further purification.

### 4.2. Apparatus

^1^H and ^13^C-NMR spectra were recorded using a Gemini 300 BB spectrometer operating at 300 MHz for ^1^H and 75 MHz for ^13^C in DMSO-d6, using TMS as internal reference. Varian 310-MS LC/MS/MS triple quadrupole mass spectrometer fitted with an electrospray ionization interface (ESI) was used. Infrared (solid ATR) spectra were recorded on a Bruker FT-IR tensor 27 spectrometer directly on small samples of the compounds in the range of 4000–400 cm^−1^. UV–Vis spectra were recorded on JASCO V-670 spectrometer in 1 cm path length quartz cuvettes. Electrochemical investigations were performed using AUTOLAB potentiostat to which three-compartment cells were coupled. The working electrode (WE) was a glassy carbon (GC) disk with a diameter of 3 mm (Metrohm, Herisau, Switzerland) bare or modified. Ag/10 mmol∙L^−1^ TBAP/CH_3_CN (0.1 M) was used as reference electrode (RE), while a platinum wire was used as auxiliary electrode (AE). The assembly of RE, WE, and AE in line was connected to the potentiostat, controlled by NOVA 2.1.4 software. All potentials were referred finally to the ferrocene/ferrocenium (Fc/ Fc^+^) redox couple.

### 4.3. Methods and Procedures

The synthesis was performed with usual equipment for organic chemistry by the general procedures, which will be described further.

The IR (solid ATR) spectra were recorded, and their parameters were noticed. The following abbreviations were used to characterize the signals: vs (very strong), s (strong), m (medium), w (weak).

For MS, air was used as drying gas at a pressure of 19 psi and temperature according to experiment. The nebulizing gas was nitrogen to 40 psi for positive ionization and air to 55 psi for negative ionization. The needle voltage had been established to the potential of 5000 V for positive ionization, and −4500 V for negative ionization. The substances were solubilized in DMSO and diluted with MeOH. The obtained solution was injected directly into the interface using a syringe pump Harvard 11PLUS with a 0.010 mL/min flow. Thus, the obtained protonated or deprotonated molecular ion was selected by the first quadrupole. Into the second quadrupole, the protonated or deprotonated molecular ion was fragmented by collision with an inert gas (argon) to 1.5 mTorr pressure. Fragments were analyzed by the third quadrupole. Prior to these experiments, the tuning of mass spectrometer was performed using PPG both for positive and negative modes.

UV–Vis spectra were recorded in DMF between 800 and 200 nm, in a quartz cuvette with a 1 cm optical path.

Electrochemical experiments for compound characterization were carried out via cyclic voltammetry (CV), differential pulse voltammetry (DPV), and rotating disk electrode voltammetry (RDE). WE, RE, and CE were immersed in the electrochemical cell containing the supporting electrolyte (0.1 M TPAP in DMF), and the curves were recorded. Each compound solution was then prepared in millimolar concentrations in TBAP/ACN or in TBAP/DMF. The voltammograms were recorded at different scan rates, starting from the stationary potential at −3 V (for cathodic scans) or +3 V (for anodic scans). DPV curves were recorded at 0.01 V/s. RDE experiments were performed at 0.01 V/s with electrode rotation rates ranging from 500 to 1500 rpm. Before each experiment, the glassy carbon electrode was properly cleaned by polishing it with diamond paste (0.25 µm) deposited on the electrode felt. Study solutions were purged of oxygen by bubbling dry nitrogen for 15 min, and the cell was then maintained at the same gas pressure throughout the experiments. At the end of the experiments, the potential was referred to as the ferrocene/ferrocenium redox couple potential (Fc/Fc^+^).

### 4.4. Computational Details

Geometry optimization of the structure was performed using B3LYP, which includes Becke’s (B3) parameter exchange functional along with Lee, Yang Parr’s (LYP) gradient corrected correlation functional [102,103,104,105,106,107] using Gaussian09 and GaussView 6.0.16 software [108,109]. Pre-geometry optimization using the molecular mechanic optimization along with forcefield implemented in the HyperChem program [110] has been performed on model structures and outputs used for further geometry optimization at the B3LYP/6-31 + G(d,p) level of theory. Vibration frequencies of the improved structures are combined using the same method to ensure that the improved buildings are compliant with the minimum space in the power area. The natural bond orbital (NBO) analyses were calculated by the NBO 3.1 module embedded in Gaussian. All frontier molecular orbital (HOMO and LUMO) isosurface maps were plotted using the Avogadro molecular editor and visualization software program [111] based on the outputs of Gaussian calculation.

## 5. Conclusions

This paper presents the results of the synthesis and properties of new tetrahydroacridine dimers:

7,7′-(ethane-1,2-diyl)*bis*(2,3-dihydro-1H-cyclopenta[b]quinoline-9-carboxylic acid) (**4a**) and 7,7′-(ethane-1,2-diyl)*bis*(1,2,3,4-tetrahydroacridine-9-carboxylic acid) (**4b**). Through this study, the range of tetrahydroacridines explored so far is extended to their dimers.

The synthesized compounds were characterized, and their structure was confirmed by spectral analyses. By DFT calculations, the main reactivity parameters were established and proved that compound **4a** is more reactive than **4b**. Compound **4a** has the lowest Eg value of 4.1834 eV compared to compound **4b** which has a higher Eg value of 4.3832 eV. This indicates that compound **4a** is less stable and more reactive than compound **4b**.

The electrochemical study allowed the evaluation of the potential at which oxidation or reduction processes take place for both compounds. In the case of compound **4a**, two non-diffusive anodic processes were highlighted, which can be attributed to the formation of film on the surface of the electrode, while at cathodic scanning reversible diffusive processes occur.

In the case of compound **4b,** the three anode processes are diffusive like the first cathodic process. The comparison between **4a** and **4b** shows that **4a** is more reactive to film formation, and it can be used in such applications. In the case of **4b**, the reduction processes could lead to the formation of non-conductive films. All these films will be examined further for protection against corrosion.

Even if their structure is quite similar, research and calculations have made it possible to highlight different properties that can be used in specific applications, such as the development of new drugs and corrosion inhibitors. The detailed examination of the curves obtained by different electrochemical investigation methods brought complementary information and allowed the establishment of the potentials of some processes with possible applications of interest: the formation of films with conductive characteristics (at 0.3V potential for compound **4a**) or insulating (at 0.6V for compound **4a**). Compound **4b** can form non-conductive films at quite negative potentials (around −2.5V).

All novelties on these compounds will be further exploited and extended in order to enlarge the field of modified electrodes for biological and corrosion applications, as recently reported [112,113].

## Figures and Tables

**Table 1:** Reactivity parameters starting from HOMO-LUMO energies of the studied compounds.

Parameter	Formula	4a	4b
Energy of the HOMO orbital (E_HOMO_), eV		−6.4722	−6.4322
Energy of the LUMO orbital (E_LUMO_), eV		−2.2888	−2.0490
Energy between HOMO and LUMO (Eg), eV	E_HOMO_ − E_LUMO_	4.1834	4.3832
Ionization potential (I), eV	I = −E_HOMO_	6.4722	6.4322
Electron affinity (A), eV	A = −E_LUMO_	2.2888	2.0490
Electronegativity (χ), eV	χ = (I + A)/2	4.3805	4.2406
Hardness (η), eV	η = (I − A)/2	2.0917	2.1916
Softness (σ), (eV)^−1^	σ = 1/η	0.4780	0.4562
Chemical potential (µ), (eV)^−1^	μ= −((I + A)/2)	−4.3805	−4.2406

**Table 2:** NBO data for compound **4a**.

Donor (ἰ)	Acceptor (j)	E^(2)^ (Kcal/mol)	E_(j)_ − E_(ἰ)_ (a.u)	F_(ἰ,j)_ (a.u)
σC_42_–H_50_	σ*C_37_–H_43_	75,640.17	6.26	19.549
σO_55_–H_56_	σ*C_29_–C_34_	14,509.10	0.05	0.785
σC_42_–H_50_	σ*C_40_–C_45_	13,898.42	16.14	13.464
πC_29_–C_31_	σ*C_40_–H_45_	13,437.61	17.48	9.427
σC_1_–H_7_	σ*C_39_–H_54_	11,842.02	0.11	1.011
σC_29_–C_31_	σ*C_34_–O_55_	11,526.30	7.97	8.798
σC_42_–H_50_	σ*C_34_–O_55_	10,631.21	16.64	12.063
σC_42_–H_50_	σ*O_57_–H_58_	8913.69	13.06	9.702
πC_29_–C_31_	σ*O_57_–H_58_	7806.22	4.40	5.512
σC_42_–H_49_	σ*C_29_–C_34_	5097.24	0.28	1.073

**Table 3:** NBO data for compound **4b**.

Donor (ἰ)	Acceptor (j)	E^(2)^ (Kcal/mol)	E_(j)_ − E_(ἰ)_ (a.u)	F_(ἰ,j)_ (a.u)
σC_32_–O_33_	σ*C_42_–C_43_	8691.80	0.08	0.760
σC_34_–O_63_	σ*C_39_–H_59_	8113.54	0.03	0.439
σC_34_–O_35_	σ*C_37_–H_46_	3609.32	0.01	0.188
σC_32_–O_33_	σC_34_–O_35_	2771.92	0.23	0.714
σC_32_–O_33_	σ*C_63_–H_64_	2544.73	0.37	0.0869
σC_32_–O_33_	σ*C_37_–H_46_	1734.11	0.32	0.663
σC_34_–O_35_	σ*C_39_–H_59_	499.24	0.24	0.3112
σC_34_–O_35_	σ*C_63_–H_64_	401.63	0.07	0.147
σC_29_–C_34_	σ*C_44_–H_45_	390.80	0.57	0.423
σC_42_–H_49_	σ*C_44_–H_45_	313.94	0.43	0.327

**Table 4:** DPV peak potentials vs. Fc/Fc^+^ for **4a** in 2 mM solution in 0.1 M TBAP/DMF.

Parameter\Peak	a1	a2	c1	c2	c3	c4
Ep (V)	0.6	0.9	−1.8	−2.3	−2.5	−2.9

**Table 5:** DPV peak potential values vs. Fc/Fc^+^ for **4b**.

Parameter\Peak	a1	a2	a3	c1	c2	c3
Ep (V)	0.303	0.565	0.767	−2.025	−2.466	−2.851

**Table 6:** Variation of current (in A) with the scan rate v (in V/s) or v1/2 (in V1/2/s1/2) for the peaks a1, a2, a3, and c1.

Parameter	a1	a2	a3	c1
Slope for i = a + b·v	1.303·10^₋5^	2.984·10^₋5^	4.969·10^₋5^	−3.235·10^₋5^
Pearson’s r for i = a + b·v	0.989	0.987	0.994	0.989
Slope for i = a + b·v ^1/2^	1.238·10^₋5^	2.841·10^₋5^	4.692·10^₋5^	−3.058·10^₋5^
Pearson’s r for i = a + b·v ^1/2^	0.999	0.9997	0.999	0.995

## Data Availability

Data are contained within the article and Supplementary Materials.

## Supplementary Materials

The following supporting information can be downloaded at: https://www.mdpi.com/article/10.3390/molecules29174082/s1, Figure S1: UV–Vis spectra obtained for different concentrations of **4b** (a), and the calibration plots at different wavelengths for **4b** (b) in DMF; Figure S2: UV–Vis spectra obtained for different concentration of **4b** (a), and the calibration plots at different wavelengths for **4b** (b) in DMF; Table S1: Main maximum wavelengths and equations for linear dependences of absorbance on concentration for **4a** and **4b** in DMF.
