# Supplementary material for: Experiments and Calculation on New N,N-bis-Tetrahydroacridines
Source: Molecules. 2024 Aug 28;29(17):4082. doi: 10.3390/molecules29174082 (PMC11396808; doi:10.3390/molecules29174082)
Supplement: Supplementary file 1 [file molecules-29-04082-s001.zip › molecules-3087187-supplementary.pdf]

Supplementary Materials

Experiments and calculation on new N,N-*bis*-tetrahydroacridines

Madalina-Marina Hrubaru[1,2], Constantin Draghici[2], Francis AurelienNgounoueKamga[3], Elena Diacu[2,*], ThankGod C. Egemonye[5], Anthony C. Ekennia[6], Eleonora-MihaelaUngureanu[2,*]

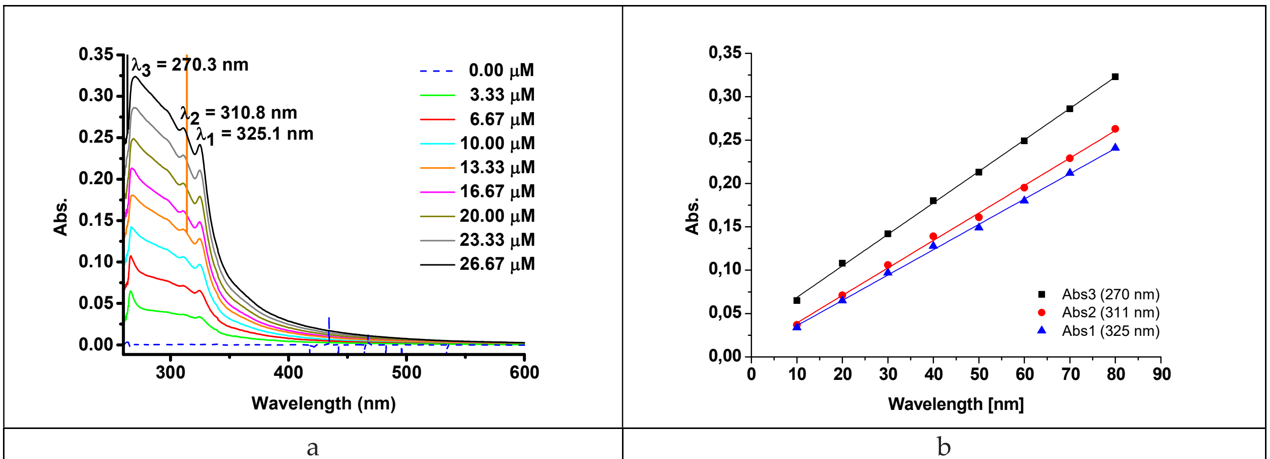

a	b

Figure S1. UV-Vis spectra obtained for different concentration of **4a** (a), and the calibration plots at different wavelengths for **4a** (b) in DMF.

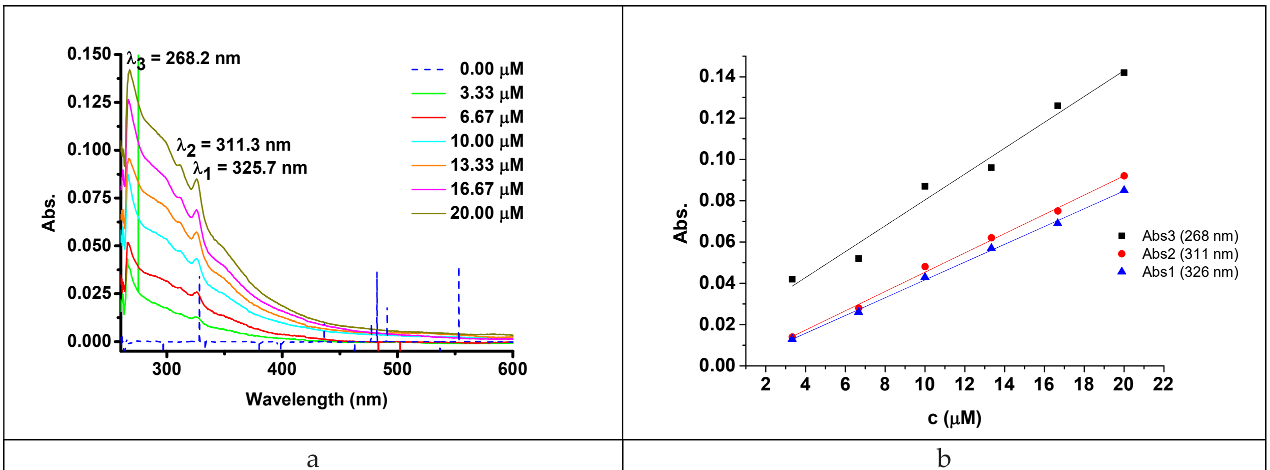

a	b

Figure S2. UV-Vis spectra obtained for different concentration of **4b** (a), and the calibration plots at different wavelengths for **4b** (b) in DMF.

Table S1. Main maximum wavelengths and equations for linear dependences of absorbance on concentration for **4a** and **4b** in DMF .

**Compound**	**λ_{max} [nm]**	**A *.vs.* C(µM) Equation**	**Pearson's R**	**ε [M^{-1}•cm^{-1}]**
**4a**	270	Abs3 = 0.0324 + 0.0109·C	0.9997	12684
	311	Abs2 = 0.0076 + 0.0095·C	0.9991	9948
	325	Abs1 = 0.0068 + 0.0088·C	0.9993	9150
**4b**	268	Abs3 = 0.0177 + 0.0063·C	0.9899	7526
	311	Abs2 = -0.0013 + 0.0047·C	0.9984	4622
	326	Abs1 = -0.0015 + 0.0043·C	0.9991	4233
